# A Hybrid Ensemble Equilibrium Optimizer Gene Selection Algorithm for Microarray Data

**DOI:** 10.3390/biomimetics10080523

**Published:** 2025-08-10

**Authors:** Peng Su, Yuxin Zhao, Xiaobo Li, Zhendi Ma, Hui Wang

**Affiliations:** School of Computer Science and Technology, Zhejiang Normal University, Jinhua 321004, China; supeng3@chinatowercom.cn (P.S.); zyx735525860@163.com (Y.Z.); mazhendi@zjnu.edu.cn (Z.M.); hwang@zjnu.cn (H.W.)

**Keywords:** gene selection, redundancy and complementarity, equilibrium optimizer, Gaussian Barebone, gene pruning strategy

## Abstract

As modern medical technology advances, the utilization of gene expression data has proliferated across diverse domains, particularly in cancer diagnosis and prognosis monitoring. However, gene expression data is often characterized by high dimensionality and a prevalence of redundant and noisy information, prompting the need for effective strategies to mitigate issues like the curse of dimensionality and overfitting. This study introduces a novel hybrid ensemble equilibrium optimizer gene selection algorithm in response. In the first stage, a hybrid approach, combining multiple filters and gene correlation-based methods, is used to select an optimal subset of genes, which is achieved by evaluating the redundancy and complementary relationships among genes to obtain a subset with maximal information content. In the second stage, an equilibrium optimizer algorithm incorporating Gaussian Barebone and a novel gene pruning strategy is employed to further search for the optimal gene subset within the candidate gene space selected in the first stage. To demonstrate the superiority of the proposed method, it was compared with nine feature selection techniques on 15 datasets. The results indicate that the ensemble filtering method in the first stage exhibits strong stability and effectively reduces the search space of the gene selection algorithms. The improved equilibrium optimizer algorithm enhances the prediction accuracy while significantly reducing the number of selected features. These findings highlight the effectiveness of the proposed method as a valuable approach for gene selection.

## 1. Introduction

DNA microarrays are arrays of DNA fragments or oligonucleotides that are immobilized on solid supports such as glass, silicon, or plastic to create gene chips [1]. After extracting mRNA from the sample under investigation, it undergoes reverse transcription and is labeled with a fluorescent tag. This labeled cDNA is then hybridized with the DNA chip, which contains thousands of genes. Following the hybridization process, any fragments on the chip that have not undergone a binding reaction are washed away. Finally, the chip is subjected to laser confocal scanning to measure the fluorescence intensity at each spot. This measurement enables the determination of the expression levels of various genes within the sample.With the application of microarray technology in the realm of medicine, there has been an exponential surge in data within the field of cancer research [2]. This technological advancement has substantially facilitated critical clinical applications in cancer research, including molecular classification, prognostic assessment, and survival prediction [3]. Given that data obtained through microarray technology often encompasses thousands of dimensions, the dimensionality curse becomes a prevailing concern when handling such datasets. Feature selection (FS), also known as gene selection (GS) in microarray data, is a common dimensionality reduction method in machine learning. Feature selection is an important step in data preprocessing and feature engineering, aiming to select the most informative and relevant feature subset from the original feature set to improve the performance and efficiency of subsequent machine learning tasks. The primary objectives of feature selection encompass dimensionality reduction, eliminating redundant and noisy attributes, and enhancing model interpretability and generalizability. Selecting the most pertinent features can diminish the consumption of computational and storage resources while mitigating overfitting issues stemming from the curse of dimensionality [4].

Gene selection is fundamentally a combinatorial optimization problem. For a dataset with *n* types of genes, gene selection aims to identify an optimal subset from $2^{n}-1$ possible combinations. This procedure reduces the classifier’s learning complexity and assessment costs and enhances classification accuracy [5]. The significance of gene selection is that genomes typically encompass a plethora of genes, among which only a small fraction play a pivotal role in specific biological processes or disease progression. By selecting pertinent subsets of genes, complexity in research and analysis can be mitigated, thereby augmenting the capability to discover potential biomarkers or therapeutic targets. In summary, gene selection is a pivotal task in genomics research and bioinformatics analysis. Through judicious selection of gene subsets, it becomes feasible to delve into gene functionality and related biological processes, better comprehend intricate genetic mechanisms and disease evolution, and thereby provide robust support for personalized medicine and drug development endeavors [6].

Feature selection methods can be categorized into three major types: filter, wrapper, and hybrid [7]. The filter is conducted independently of feature selection and machine learning algorithms. Common filter methods include correlation coefficients, information gain, the chi-square test, etc., which assess the importance of features by calculating the correlation or independence between features and the target variable and subsequently select features with high importance scores [8,9,10,11]. Filters typically require a single evaluation and ranking of features, which are agnostic to the specific learning algorithm. Therefore, they exhibit high efficiency and broader applicability when dealing with large-scale datasets [12]. However, due to their independent assessment and ranking of each feature, these methods sometimes overlook the potentially significant information gained from combinations of relatively less essential features with others.

Consequently, filters often neglect the interrelationship among features, leading to information loss [13]. Furthermore, filters solely base their selection on the correlation between features and the target variable, without considering the performance of the ultimate learning algorithm. This may result in suboptimal performance of the chosen subset in practical learning tasks.

A wrapper method refers to incorporating the feature selection process within machine learning algorithms. Commonly employed wrapper methods include recursive feature elimination, evolutionary algorithms, iterative models, etc. These methods continuously train models and assess the importance of features based on model performance, subsequently refining the selection of the optimal feature subset [14]. Due to their adaptability, strong search capabilities, and characteristics of conciseness and flexibility, evolutionary algorithms are commonly employed as a search approach within the current framework of wrapper methods. The most frequently utilized evolutionary algorithms include the Whale Optimization Algorithm (WOA) [15], Genetic Algorithm (GA) [16], Differential Evolution Algorithm (DE) [17,18,19], Particle Swarm Algorithm (PSO) [20], Ant Colony Optimization Algorithm (ACO) [21,22], Marine Predator Algorithm (MPA) [23], Artificial Bee Colony Algorithm (ABC) [24], Slime Mould Algorithm (SMA) [25,26], Grey Wolf Optimization Algorithm (GWO) [27], Crow Search Algorithm (CSA) [28], etc. A wrapper, by employing a learning algorithm for evaluation, offers relatively higher selection accuracy to maximize the performance of learning algorithms. The wrapper can more accurately capture nonlinear relationship and interactions among features than filter methods [29]. However, due to their direct use of learning algorithms for feature subset evaluation, wrapper methods may encounter longer computation times and potential overfitting issues when selecting feature subsets.

Hybrid feature selection methods are an approach that integrates multiple feature selection techniques to enhance the accuracy, robustness, and generalization capability of feature selection. Hybrid feature selection algorithms can be categorized into three main types: those based on filtering and wrapping, based on a combination, and based on ensemble learning [30,31]. Singh et al. [32] proposed a four-step hybrid ensemble feature selection algorithm. Firstly, the dataset is partitioned using a cross-validation process. Subsequently, various weighted score-based filtering methods are integrated into the filtering step to generate feature rankings. Thirdly, the Sequential Forward Selection algorithm is employed as a wrapping technique to attain the optimal feature subset. Lastly, the obtained optimal subset is processed for subsequent classification tasks. Lai et al. [33] introduced a hybrid method named IG-ISSO, which utilizes IG as a filter to select genes with maximum information content, then uses Improved Simplified Swarm Optimization (ISSO) as a gene search engine to guide the search for the optimal gene subset. Mandal et al. [34] proposed a three-stage feature selection framework. In the first stage, a set is formed using four filtering methods. Subsequently, three classification algorithms are employed to assess each feature in the composite setting, calculating the average precision. Features with higher precision are selected, and in the final stage, the Whale Optimization Algorithm is employed to reduce the feature count further and achieve enhanced accuracy.

The combination hybrid method derives the final subset of features by combining the results of multiple feature selection algorithms. Common combination approaches include the voting method and the weighted summation method, etc. [35]. Drotar et al. [36] proposed a hybrid feature selection method based on voting aggregation schemes, such as majority voting, single transferable vote, Borda count, and a novel weighted Borda count. Yao et al. [37] introduced two feature selection methods based on ranking information: FS-MRI and SFFS-RI. These methods marked the first successful integration of undersampling techniques with the AdaBoost ensemble method. Jas et al. [38] introduced two novel hybrid feature selection methods: Metric Ranking Feature Inclusion and Accuracy Ranking Feature Inclusion. These methods follow a two-stage approach, with the first stage involving feature ranking, followed by feature subset selection.

Ensemble learning is a technique that combines multiple base learners into a strong learner. The strength of ensemble learning lies in its ability to harness the collective wisdom of multiple feature selection models, thereby enhancing the robustness and accuracy of feature selection. Wang et al. [39] proposed an ensemble feature selection framework that utilizes sampling techniques to obtain multiple sampled datasets and employs a basic feature selector to choose a feature subset from each dataset. Two aggregation strategies are applied to the basic feature subsets to merge them into a single set. Chiew et al. [40] introduced an integrated feature selection framework named HEFS, which employs the Cumulative Distribution Function gradient (CDF-G) algorithm to generate primary feature subsets. Subsequently, the framework employs function perturbation sets to derive a set of baseline features from secondary feature subsets. The most commonly used methods in ensemble learning are boosting and bagging. Ngo et al. [41] introduced Evolutionary Bagging Ensemble Learning, which employs evolutionary algorithms to evolve the contents of the bags, thereby iteratively enhancing the ensemble by providing diversity within the bags.

Equilibrium Optimizer (EO) [42] is an evolutionary algorithm introduced in 2020, inspired by the principles of balance observed in nature. It addresses single-objective and multi-objective optimization problems by emulating the equilibrium behaviors observed in physical systems. Due to its strong adaptability, high search efficiency, and convergence performance, EO has been applied in various fields, including wind farm layout [43], job scheduling [44], fuel cell modeling [45], multi-objective optimization [46], image segmentation [47], stock market prediction [48], etc. However, when faced with complex problems, the algorithm still exhibits sensitivity to initial subsets and premature convergence issues. Consequently, numerous scholars have proposed variant versions of EO to address these aspects. Zhong et al. [49] proposed an adaptive quantum-inspired feature selection algorithm based on artificial bee colony, which integrates quantum theory and adaptive mechanisms into the algorithm’s updating rules to enhance convergence. Additionally, the updating mechanism of the artificial bee colony is incorporated into the algorithm to obtain suitable feature selection solutions. Thapliyal et al. [50] proposed a hybrid optimization algorithm called ASCASO, which combines the accelerated sine-cosine algorithm and the balanced optimizer. The algorithm improves performance in solving practical problems by combining multiple variants. Agrawal et al. [51] introduced the Normalized Mutual Information-based Equilibrium Optimizer (NMIEO), which employs a localized search strategy based on normalized mutual information and utilizes chaotic mapping for population initialization, enhancing the algorithm’s search capability. Guha et al. [52] proposed a binary-adaptive Equilibrium Optimizer (DEO) for addressing binary optimization problems. They employed a sigmoidal transfer function to map continuous EO values into a binary domain and incorporated Simulated Annealing (SA) as a local search procedure, further enhancing the search capability of DEO.

This paper presents a hybrid multiple-filter ensemble EO algorithm to address the feature selection problem in high-dimensional microarray data. In the first stage, a multiple-filter ensemble approach is utilized to eliminate noise and redundant data from the dataset. Symmetric uncertainty and conditional mutual information are employed to quantify features’ redundancy and complementary relationships. The second stage utilizes an improved EO approach based on the Gaussian Barebone mechanism and the gene pruning strategy, which enhances its search capability and enables it to adapt to the complexity of the gene selection search space. To demonstrate the performance of the proposed method, a comparison is conducted against nine feature selection methods on fifteen microarray datasets. The results illustrate the superior performance of the proposed approach.

The remaining parts of the study proceed in the following way: Section 2 is concerned with the methods employed for the study. Section 3 provides a detailed explanation of the method we have proposed. Section 4 introduces the experimental design of the proposed method in detail. Section 5 gives the experimental results and a critique of the findings. The purpose of the final section is to summarize the paper and present plans for future work.

## 2. Methodology

This section mainly introduces the basic filtering methods and EO algorithm.

### 2.1. Filtering Methods

#### 2.1.1. Fisher Score

The Fisher score, proposed by Gu et al. [53], is a statistical method widely employed in machine learning and pattern recognition. It is based on Fisher discriminant analysis, which evaluates the significance of features by measuring the differences between different categories within a dataset. The computation of the Fisher score assesses the importance of features by quantifying the dissimilarities among distinct classes in the dataset. The Fisher score of feature $f_{i}$ can be expressed using the following formula:

(1) $$F\left(f_{i}\right)=\frac{\sum_{k=1}^{c}n_{k}{\left(u_{f_{i}}^{k}-u_{f_{i}}\right)}^{2}}{\sum_{k=1}^{c}n_{k}{\left(\sigma_{f_{i}}^{k}\right)}^{2}}$$

where $u_{f_{i}}$ represents the average value, and $\sigma_{f_{i}}^{k}$ corresponds to the standard deviation in class *k* and feature $f_{i}$. $n_{k}$ denotes the number of samples in class *k*.

#### 2.1.2. ReliefF

ReliefF [54] is a feature weight-based algorithm that handles both continuous and discrete attributes, aiming to select the most discriminative features from a given dataset. It relies on a heuristic algorithm to evaluate the importance of features based on their contribution to the target variable. The core idea of the algorithm lies in estimating the significance of features by comparing the nearest neighbors among samples.

#### 2.1.3. Chi-Square

The chi-square test [55] is a statistical method used to assess the deviation between observed and expected frequencies to determine if the observed data aligns with a theoretical model. It is commonly employed for comparing differences between observed and expected values to ascertain the presence of a correlation between two variables. The chi-square test calculates the chi-square statistic by comparing observed and expected frequencies and then determines the significance of the differences based on degrees of freedom and the chosen significance level. Where $N_{ij}$ represents the number of instances of $C_{i}$ in the *j*th interval, and $M_{ij}$ denotes the total number of instances in the jth interval, the expected value of $N_{ij}$ is as follows:

(2) $$E_{ij}=\{\frac{M_{ij}*\left|C_{i}\right|}{N}\}$$

The calculation formula for chi-squared is as follows, where I represents the number of intervals:

(3) $$X^{2}=\sum_{i=1}^{c}\sum_{j=1}^{I}\frac{\left(N_{ij}-E_{ij}\right)}{E_{ij}}$$

#### 2.1.4. Pearson Correlation Coefficient

The calculation of the Pearson correlation coefficient (PCC) [56] is based on the covariance of the observed data and the standard deviations of each variable. It measures the strength of the linear relationship between two variables and how one variable changes in relation to another variable.

The formula for calculating the Pearson correlation coefficient is as follows:

(4) $$\rho =\frac{\sum \left(X_{i}-\bar{X}\right)\left(Y_{i}-\bar{Y}\right)}{\sqrt{\sum {\left(X_{i}-\bar{X}\right)}^{2}\sum {\left(Y_{i}-\bar{Y}\right)}^{2}}}$$

where ρ represents the Pearson correlation coefficient, $X_{i}$ and $Y_{i}$ denote the observed values of the two variables, and $\bar{X}$ and $\bar{Y}$ represent their respective means. The summations are performed over the entire dataset. When the coefficient is positive, it indicates a positive correlation between two variables, meaning that an increase in one variable accompanies an increase in the other variable. Conversely, when the coefficient is negative, it suggests a negative correlation between the variables, implying that a decrease in one variable accompanies an increase in the other variable. The closer the correlation coefficient is to 1 or −1, the stronger the correlation between the variables.

#### 2.1.5. Neighborhood Component Analysis

Neighborhood Component Analysis (NCA) [57] is a supervised dimensionality reduction method that aims to learn a linear transformation by optimizing an objective function. Its goal is to make instances within the neighborhood of each sample easier to distinguish in the new lower-dimensional representation. NCA is utilized for feature selection by optimizing an objective function to learn a linear transformation matrix. The objective function aims to maximize the discriminability of neighbor pairs, thereby identifying a subset of features that significantly impact the given task. By calculating pairwise distances, determining neighbors, defining the objective function, conducting the optimization process, and sorting features, NCA can identify features with higher weights to enhance the predictive performance of classification or regression tasks.

### 2.2. Equilibrium Optimizer

The Equilibrium Optimizer (EO) is a heuristic optimization algorithm inspired by the equilibrium points in dynamic systems and the potential energy in Hamiltonian mechanics. This algorithm aims to solve optimization problems by simulating the process of reaching equilibrium in dynamic systems. The strong search performance of EO can be attributed to three crucial mechanisms: initialization, balance-pool, and concentration-update stages.

Step 1: Initialization. In the initialization stage, the positions of each particle are randomly generated throughout the entire search space:

(5) $$X_{i}=l_{b}+r\left(u_{b}-l_{b}\right)$$

where $X_{i}$ is the initial position of the particle, $u_{b}$ is the upper bound of the search space, *r* is a random number from 0 to 1, and $l_{b}$ is the lower bound of the search space.

Step 2: Construction of the equilibrium pool. To accelerate the particles’ search towards the optimal solution, EO selects the positions of the four best particles discovered so far based on their fitness values and places them into the equilibrium pool after each iteration. Furthermore, to enhance particle diversity, the average position of the aforementioned four types of particles is calculated and stored in the equilibrium pool. The equilibrium pool is defined as follows:

(6) $$X_{\mathrm{eq},\mathrm{POOL}}=\left\{\begin{array}{c} X_{\mathrm{eq}(1)},\;X_{\mathrm{eq}(2)},\\ X_{\mathrm{eq}(3)},\;X_{\mathrm{eq}(4)},\;X_{\mathrm{eq}(\mathrm{ave})} \end{array}\right\}$$

where $X_{eq,POOL}$ represents the equilibrium pool, $X_{eq(i)}$i=(1,2,3,4) represents the position of the best four particles discovered so far, and $X_{eq(ave)}$ represents the average position of the four best particle positions, expressed as

(7) $$X_{\mathrm{eq}(\mathrm{ave})}=\frac{X_{\mathrm{eq}(1)}+X_{\mathrm{eq}(2)}+X_{\mathrm{eq}(3)}+X_{\mathrm{eq}(4)}}{4}$$

At the beginning of each iteration, a random particle is selected as the best particle in the equilibrium pool, which significantly increases the diversity of the population due to random selection.

**Figure 1:**
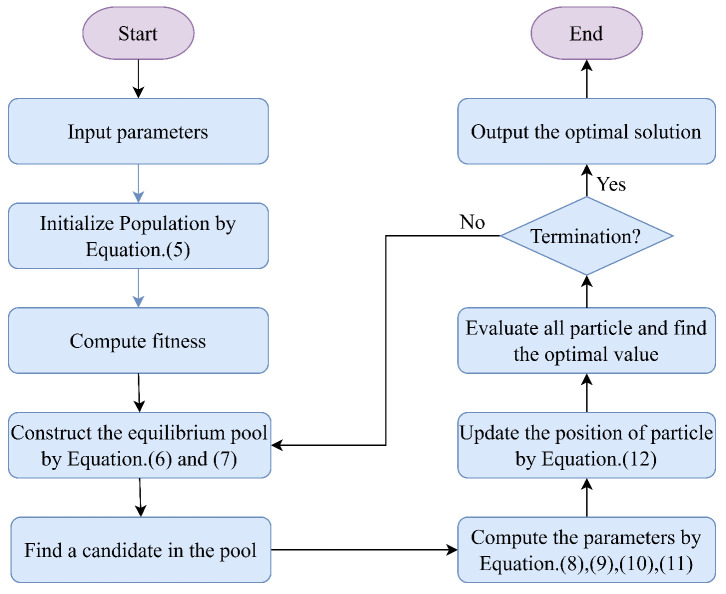
The flowchart of EO. The complete process of EO is divided into the following key stages. Initialization: Randomly generating an initial population, setting parameters. Fitness evaluation: Calculating the fitness value of each solution to measure quality. Balance factor update: Dynamically adjusting the balance factor of EO to optimize the weights between exploration and exploitation. Spatial position update: Iteratively improving solutions through “exponential term” and “generation rate” strategies. Termination condition check: If the maximum number of iterations or convergence threshold is reached, output the optimal solution; otherwise, return the fitness evaluation.

Step 3: Position update. EO consists of two rules in the update of the position of the particles: The concentration-update rule, controlled by an exponential term (F), and the equilibrium-state rule, controlled by the generation rate (G) [58,59]. The exponential term (F) controls the balance between exploration and exploitation in the algorithm and can be expressed as

(8) $$F=a_{1}sigh\left(r_{1}-0.5\right)\left[\exp\left(-r_{2}t\right)-1\right]$$

where $a_{1}$ is set as a constant value of 2 to control the exploration capability of the algorithm [42], $r_{1}$ and $r_{2}$ are random numbers between 0 and 1, and *t* is the coefficient of EO that receives an update in each iteration, which can be expressed as

(9) $$t={\left(1-T/M_{iter}\right)}^{\left(a_{2}T/M_{iter}\right)}$$

where *T* represents the current iteration of EO, $M_{iter}$ represents the maximum number of iterations, and $a_{2}$ is a constant that controls the exploitation capability of EO. If $a_{1}$ is larger, the algorithm will have stronger exploration capability, and similarly, if $a_{2}$ is larger, the algorithm will have stronger exploitation capability. Typically, these two values are set to 2 and 1, respectively. The generation rate (G) is another important update rule in EO, and it is defined as

(10) $$G=-P\left(X_{eq}-r_{2}X\left(T\right)\right)F$$

(11) $$p=\left\{\begin{array}{c} 0.5\times {\;r}_{d1}\times u\;\;\;\;\;\;\;\;\;\;\;\;\;r_{d2}\geq GP\\ \;0\;\;\;\;\;\;\;\;\;\;\;\;\;\;\;\;\;\;\;\;\;\;\;\;\;r_{d2}<GP \end{array}\right.$$

where $X_{eq}$ is the best particle selected from the equilibrium pool in the current iteration, X(T) represents the position of the particles in the current iteration, $r_{d1}$ and $r_{d2}$ are random numbers between 0 and 1, *u* is a unit vector, and GP is set to 0.5. Therefore, the update rule for particles in the EO algorithm can be expressed as

(12) $$\begin{array}{cc} X(T+1) & =X_{\mathrm{eq}}+\left(X\left(T\right)-X_{\mathrm{eq}}\right)F\\ \; & \;+\frac{(1-F)G}{r_{3}\;V} \end{array}$$

where X(T+1) represents the updated position of the particle, X(T) represents the position in the previous iteration, $r_{3}$ is a random vector between 0 and 1, and *V* is the unit vector. After each iteration, the particle positions are checked for boundary violations, and the fitness value for each particle is calculated. Then, the positions of particles in the equilibrium pool are updated accordingly. Figure 1 shows the flowchart of the algorithm.

## 3. The Proposed Method

To address the characteristics of large search spaces and abundant redundancy in high-dimensional data, this paper proposes a hybrid multi-filter ensemble feature selection method based on EO, named RCMF-GBGPSEO. This section provides a detailed description of the proposed method in two stages.

### 3.1. Stage 1: Multi-Filter Ensemble Strategy Based on Redundancy and Complementarity

**Figure 2:**
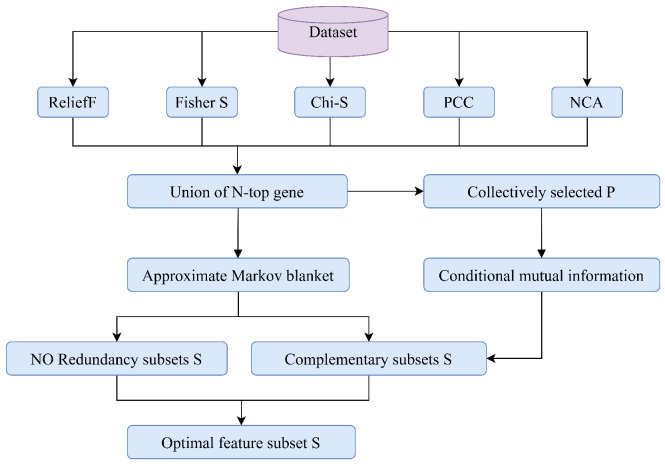
The flowchart of RCMF. Exploiting symmetric uncertainty and conditional mutual information to measure redundancy and complementarity between genes, and combining them with five different filters to form a novel filtering method.

In this part, we utilize symmetric uncertainty and conditional mutual information to measure the redundancy and complementarity between genes and combine them with five different filters to form a novel filtering method. Figure 2 shows the flowchart of the RCMF.

#### 3.1.1. Symmetric Uncertainty

Symmetric uncertainty (SU) [60] is a concept in information theory used to measure the correlation between two random variables. It is derived from the notion of entropy and can be employed to quantify the uncertainty of one variable given another variable. The SU is obtained by calculating the entropy of these two variables and their joint entropy. Entropy represents the amount of information or uncertainty in a random variable, so higher entropy indicates higher uncertainty in the variable. The SU is computed to assess the correlation between features and the target variable, selecting the most informative features. Furthermore, symmetric uncertainty can also measure the similarity between two sets. We can quantify their differences and similarities by calculating the symmetric uncertainty of two sets. Given this, it is necessary to analyze the nonlinear correlation between features based on entropy-based measures. Treating each feature as a random variable, we utilize entropy to compute their uncertainty. The calculation of entropy H(x) is as follows:

(13) $$H\left(X\right)=-\sum_{i\in X}P\left(x_{i}\right)\log_{2}P\left(x_{i}\right)$$

where P(x) represents the probability distribution of all features *x*. The mathematical formula for SU typically employs the information gain ratio (IGR) in its calculation.

(14) $$IG\left(X,Y\right)=H\left(X\right)-H\left(X|Y\right)$$

where IG(X,Y) represents the information gain of *X* with respect to *Y*, H(X) denotes the entropy of *X*, H(Y) represents the entropy of *Y*, and H(X|Y) represents the conditional entropy of *X* given *Y*. The conditional entropy indicates the uncertainty of *X* when *Y* is known. Therefore, the formula for SU is as follows:

(15) $$SU\left(X,Y\right)=2*\frac{IG(X,Y)}{H(X)+H(Y)}$$

SU measures the correlation between *X* and *Y* by calculating the information gain ratio. It ranges from 0 to 1, where a higher value indicates a stronger correlation between *X* and *Y*.

#### 3.1.2. Mutual Information and Conditional Mutual Information

Mutual information (MI) is widely used in information theory to measure the correlation and shared information between two random variables. It provides a measure to determine the dependence and information transfer between variables. The mathematical definition of MI is as follows:

(16) H(X,Y)=H(X)+H(Y|X)

(17) I(X;Y)=H(X)+H(Y)−H(X,Y)

where I(X,Y) represents the (MI) between the variables *X* and *Y*, H(X) and H(Y) represent the entropy of the variables *X* and *Y*, respectively, and H(X,Y) represents the joint entropy of the variables *X* and *Y* (a measure of uncertainty shared by the two variables). H(Y|X) is the conditional entropy; the conditional uncertainty of *X* under the given condition *Y* can be expressed by the conditional probability distribution p(x|y), for which the mathematical formula is as follows:

(18) $$H\left(X|Y\right)=-\sum_{y\in Y}p\left(y\right)\sum_{x\in X}p\left(x|y\right)\log p\left(x|y\right)$$

Conditional mutual information (CMI) is a concept derived from the notion of MI. It measures the correlation between two random variables while considering the influence of one variable given the other variable as a condition. It is widely employed in information theory and statistics to study the dependency relationships and information transmission between variables. MI measures the shared information between two random variables, whereas CMI further considers the scenario when a conditional variable is given. The mathematical definition of CMI is as follows:

(19) CMI(X;Y|Z)=H(X|Z)−H(X|Y,Z)

CMI(X;Y|Z) represents the conditional mutual information between variables *X* and *Y* given condition variable *Z*. H(X|Z) represents the conditional entropy of variable *X* given condition variable *Z*. H(X|Y,Z) represents the conditional entropy of variable *X* given variables *Y* and *Z*.

CMI can be used to study the dependency relationship between variables. It quantifies the shared information between variables *X* and *Y* given a set of known condition variables *Z*. If the conditional mutual information is zero, it means that there is no additional correlation between variables *X* and *Y* given the condition variables *Z*. On the other hand, a non-zero CMI indicates the presence of meaningful correlation between variables *X* and *Y* given the condition variables *Z*.

#### 3.1.3. The Proposed Strategy: RCMF

In GS, the optimal subset often contains strongly correlated features and weakly correlated features, whereas the weakly correlated features include only relevant and non-redundant features. Since filters only consider the relationship between individual features and the label, aggregating the output of multiple filters often includes redundant features. This study used five mainstream filters (Fisher Score, ReliefF, chi-square, Pearson Correlation Coefficient, and Neighborhood Component Analysis). The previous section mentioned that SU can effectively measure the correlation between features and the label. Therefore, in this stage, an approximate Markov blanket proposed by Koller et al. [61] was used to remove redundant features while aggregating multiple filters.

Current research typically advocates the removal of redundant genes; however, this approach is not suitable for biological data. The simultaneous selection of two correlated features may yield additional information [62]. For example, a set of genes may interconnect to produce downstream proteins. Therefore, complementarity is often a neglected aspect in GS and is of significant importance in improving the accuracy of feature selection. The concurrent selection of two correlated features leads to additional information generation. In the preceding discussion, we elucidated the concepts of MI and CMI. While MI quantifies the interdependence between two characteristics, CMI gauges the association between a characteristic and the label when conditioned on another characteristic. Consequently, this research employs CMI as the criterion for assessing the presence of complementarity between two features. With regard to correlation, the focus lies on examining the interrelationship between two features. Given the expansive span of the original feature space, in this article, the features selected by all five filters are regarded as strongly correlated features. We search for features related to them in the feature space pruned using SU, thereby improving the diversity of features. We show the pseudo-code of the RCMF method as Algorithm 1.
**Algorithm 1** The pseudo-code for RCMF**Input:** Feature set $F=\{f_{1},f_{2},f_{3},...,f_{n}\}$ representing the set of features consisting of the N-top features selected by the five filters, set $P=\{p_{1},p_{2},p_{3},...,p_{l}\}$ representing the set of features collectively selected by the five filters, set S=∅, and class *C*. **Output:** The selected feature subset *S* **for** i=1 to *n* **do**     **for** j=i+1 to *n* **do**         calculate $SU_{i,j}$, $SU_{i,j}$, and $SU_{j,c}$         **if** $SU_{i,c}\geq SU_{j,c}$ and $SU_{i,j}\geq SU_{j,c}$ **then**            continue         **else**            add $f_{j}$ into set *S*←$S=S+\{f_{j}\}$         **end if**     **end for** **end for** $F=\{f_{1},f_{2},f_{3},...,f_{m}\}$←F=F−S **for** i=1 to *l* **do**     **for** j=1 to *m* **do**         calculate $I(f_{j},C|p_{i})$         **if** I<0 **then**            continue         **else**            add $f_{j}$ into set *S*←$S=S+\{f_{j}\}$         **end if**     **end for** **end for** **return** *S*

### 3.2. Stage 2: Equilibrium Optimizer Based on Gaussian Barebone Mechanism and Gene Pruning Strategy (GBGPSEO)

#### 3.2.1. Improved Gaussian Barebone Mechanism

As mentioned earlier, in the later iterations, EO algorithms still suffer from drawbacks such as poor convergence and susceptibility to local optima. Therefore, choosing an appropriate mutation strategy to help escape local optima is crucial for improving EO algorithms. The Gaussian Barebone (GB) [63] strategy is a method based on the Gaussian distribution, which has the advantage of being simple and easy to implement. It can assist the population in evolving toward the optimal direction, thus continuously approaching the optimal solution. The Cauchy distribution is another probability distribution function, with a larger step size than the Gaussian distribution. It can assist a population undergoing larger-scale mutations, thereby increasing the likelihood of the algorithm escaping from local optima.

In this study, a mutation parameter CR is used to guide the direction of population mutation. When the generated random probability is less than CR, the Gaussian distribution is used to guide the update strategy of individuals. Conversely, when it is greater than or equal to CR, the Cauchy mutation is employed to update the positions of individuals. In this approach, an adaptive CR is utilized to adjust the evolutionary strategy of the population. This allows the algorithm to use a larger mutation range with Cauchy mutation in the early iterations and gradually converge towards the optimal solution using Gaussian mutation in later stages. The strategy of this method is as follows:

(20) $$CR={CR}_{max}-\left({CR}_{max}-{CR}_{min}\right)\left(T/M_{iter}\right)$$

(21) $$V_{i,j}=\left\{\begin{array}{cc} \begin{array}{c} G(\frac{X_{\mathrm{Leader}}-X_{i,j}}{2},\\ \;\left|X_{\mathrm{Leader}}-X_{i,j}\right|), \end{array}\; & r<\mathrm{CR}\\ X_{i,j}+k\left(X_{1,j}-X_{2,j}\right),\; & r\geq \mathrm{CR} \end{array}\right.$$

In this equation, CR represents the mutation parameter, $CR_{max}$ denotes the maximum value of the mutation parameter, and $CR_{min}$ represents the minimum value of the mutation parameter. $V_{ij}$ represents the position of the ith individual in the jth dimension. $X_{i,j}$ represents the current position of the ith individual in the jth dimension, while $X_{Leader}$ represents the current best individual obtained from the equilibrium pool. *k* corresponds to the Cauchy distribution value at the position $X_{i,j}$, and $X_{1,j}$ and $X_{2,j}$ represent the values of two different individuals of dimension *j*. *r* is a random number between 0 and 1.

#### 3.2.2. MRMR-Based Gene Pruning Strategy

Although the EO has demonstrated strong search capabilities, its ability to search in the optimal region is still insufficient. Therefore, it is necessary to combine it with a local search strategy. According to ref. [64], the gene pruning strategy (GPS) is a powerful local search strategy that can retain the most information while reducing the size of the gene set, thereby achieving higher classification accuracy. GPS is achieved through the gene pruning probability (GPP), and this strategy is executed when the pruning probability ρ is less than the GPP. Specifically, gene pruning removes redundant or low-contribution genes based on a fitness score threshold (e.g., genes with scores below 0.3 are discarded). This ensures computational efficiency while preserving key genetic diversity. Starting from a solution, GPS sequentially prunes genes in a certain order. If the pruned solution does not affect the classification accuracy, the gene is removed; otherwise, it is retained. The order is determined based on MRMR, where less important genes are prioritized for removal from the solution. Algorithm 2 gives the pseudo-code of the MRMR-based gene pruning strategy.
**Algorithm 2** The pseudo-code of the MRMR-based gene pruning strategy**Input:** A solution $X=\{x_{1},x_{2},x_{3},\ldots ,x_{n}\}$, ρ, GPP **Output:** The solution $X=(x_{1},x_{2},x_{3},\ldots ,x_{n})$ **if** ρ<GPP **then**     Use MRMR with the selected genes in *X* to produce a ranking list $L=\{l_{1},l_{2},l_{3},\ldots ,l_{n}\}$.     **for** i=1 to *n* **do**         Prune the $l_{i}$ from the solution *X* and let $X^{*}=X$.         **if** $F\left(X^{*}\right)<F\left(X\right)$ **then**            Let $X=X^{*}$.         **end if**     **end for** **end if** **return** *X*

### 3.3. Overall Overview of the Proposed Method (RCMF-GBGPSEO)

**Figure 3:**
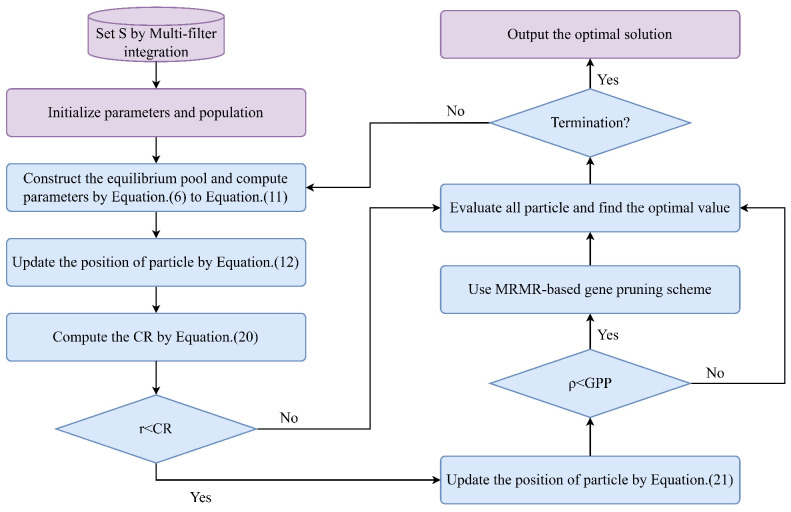
The flowchart of the proposed method. Compared to the original EO algorithm, GBGPSEO incorporates an enhanced Gaussian perturbation mechanism and genetic pruning strategy. The Gaussian perturbation mechanism can generate massive mutations to assist the algorithm in escaping local optima, while genetic pruning can strengthen the algorithm’s convergence capability.

In stage 1, the method utilizes an ensemble of multiple filters to reduce the size of the search space. This integration takes into account gene redundancy while also considering gene complementarity, thereby enhancing gene diversity and improving the accuracy of subsequent gene selection. In stage 2, the method employs the improved GB and GPS to enhance the search capability of the EO algorithm and improve its convergence performance in the vicinity of local optima during later iterations. Figure 3 shows the flowchart of the proposed method.

## 4. Experiment

### 4.1. Datasets

To test the performance of the proposed method, 15 datasets were used in the experiments. These included 13 high-dimensional biomedical datasets obtained from the microarray data repository [65], as well as three high-dimensional datasets obtained from UCI data to validate the stability and accuracy of the proposed method. The features of these datasets are summarized in Table 1 and Table 2.

### 4.2. Performance Metrics

In this study, we primarily focus on the performance of the proposed method in two stages. In stage 1, to evaluate the performance of the multi-filter ensemble, we primarily focus on its stability. We use the Jaccard index to measure the stability of the obtained gene subset through filtering [66,67]. The formula for calculating the Jaccard index is as follows:

(22) $$Jaccard\left(S\right)=\frac{2}{Q\left(Q-1\right)}\sum_{i=1}^{Q-1}\sum_{j=j+1}^{Q}\frac{\left|S_{i}\cap S_{j}\right|}{\left.{\left|S\right.}_{i}\cup S_{j}\right|}$$

We use Q-fold cross-validation on the original dataset with the ensemble filtering strategy to obtain Q gene subsets $S_{i}$,(i=1,,,Q), and then calculate the Jaccard index.

Microarray datasets are typically high-dimensional and imbalanced. The G-mean metric measures the balance performance of a learning algorithm between two classes [68,69]. Additionally, AUC indicates the trade-off between the actual positive rate (TP) and false positive rate (FP) across the entire sample distribution of the dataset. In this study, the proposed ensemble filtering strategy is experimentally evaluated using both the G-mean and AUC metrics to assess the performance of the filtering method. The formulas for these metrics are as follows:

(23) $$G-mean=\sqrt{\frac{TP}{TP+FN}\times \frac{TN}{TN+FP}}$$

For multi-class classification problems, the G-mean can be expressed as

(24) $$G-mean={\left(\prod_{i=1}^{k}\frac{{TP}_{i}}{{size}_{i}}\right)}^{\frac{1}{k}}$$

where TP represents true positive, FP represents false positive, FN represents false negative, and TN represents true negative. $TP_{i}$ denotes the number of samples correctly classified as belonging to class *i*. $size_{i}$ represents the number of samples belonging to class *i*. *k* represents the number of classes.

(25) $$AUC=1+\frac{{TP}_{rata}-{FP}_{rata}}{2}$$

For multi-class classification problems, the mean area under the curve (MAUC) is used as a representation.

(26) $$MAUC=\frac{\sum_{i=1}^{k}{AUC}_{i}}{k}$$

To measure the performance of the GBGPSEO algorithm proposed in the second stage, we use the following metrics: average classification accuracy (MeanA), average subset size (MeanS), average fitness value (MeanF), and the standard deviation (std) of all these metrics. The fitness function of the algorithm is defined as follows:

(27) $$Fitness=\alpha E\left(s\right)+\left(1-\alpha \right)\frac{\left|N-R\right|}{\left|N\right|}$$

where α is a value between 0 and 1, we set it to 0.9 in this study. E(s) denotes the average error rate, and ten-fold cross-validation is employed to prevent overfitting. Accuracy assessment is conducted using a KNN classifier with K = 5. *N* refers to the size of the gene subset obtained in the first stage, while *R* signifies the size of the gene subset obtained by the wrapper algorithm.

### 4.3. Comparison Algorithms and Parameter Settings

The GBGPSEO algorithm proposed in this paper was compared with the original EO algorithm [42] and eight state-of-the-art swarm intelligence algorithms (oBABC [70], PSO [71], WOA [72], SMA [73], HOA [74], MPA [75], GNDO [76], MRFO [77]). Both the scenarios with an integrated filtering strategy and unfiltered data were considered. Following reference [78], the threshold for RCMF is set at 100. The parameter settings for the algorithms are shown in Table 3. All algorithms were implemented on an Intel(R) Core(TM) i5-10200H CPU with 16 GB RAM using Matlab (R2021b). To ensure the reliability of the experiments, the population size for all algorithms was set to 10, and the number of iterations was set to 100.

## 5. Results and Discussion

This section provides experimental evidence to demonstrate the advantages of the proposed method from three aspects. For the integration filtering strategy in the first phase, we used the Jaccard index to illustrate the stability of the proposed strategy. We measured its performance using the G-mean and AUC. As for the proposed GBGPSEO algorithm, we compared it with eight swarm intelligence algorithms. We conducted the comparison using both filtered and unfiltered approaches and performed 20 experiments for each dataset.

### 5.1. The Stability of RCMF

To validate the performance of the proposed integrated multi-filter strategy based on redundancy and complementarity, this section primarily investigates the stability of gene sets obtained through filtering using RCMF. The stability of the filtering strategy must be maintained to provide a consistent search space for subsequent wrapper methods, thereby ensuring their performance. In this experiment, we employed six different threshold values with an interval of 50. The strategy was applied six times for each threshold value to perform filtering. After obtaining six distinct gene sets, the Jaccard index was computed for each pair of gene sets.

Table 4 illustrates the stability of the proposed RCMF across various filtering thresholds. As depicted in the table, across six different thresholds ranging from 50 to 300, the proposed approach demonstrates strong stability across three UCI datasets and 12 microarray datasets. Except for the DBWorld dataset at a threshold of 300, which shows a Jaccard index of 0.998, all other Jaccard index values are 1. Therefore, through the Jaccard index, we can observe that the proposed method consistently filters out redundant and irrelevant features at different thresholds, thereby achieving a robust gene set.

### 5.2. Comparison of RCMF Against Single Filters

This section compares the classification performance of RCMF with five different filtering methods: ReliefF, Fisher score, chi-square, PCC, and NCA. The comparison is based on two metrics: G-mean and AUC. To investigate the impact of RCMF on the filtering process, we compared RCMF with individual filters on filtered gene subsets. A 5-fold cross-validation was performed for each filter method, and the performance was evaluated using two metrics, G-mean and AUC, across 15 datasets.

Table 5 presents the comparison results of RCMF with individual filters in terms of G-mean and AUC. Among the 15 datasets, RCMF achieved the best results on five datasets. On nine datasets, other individual filters obtained the best results alongside RCMF. On the Colon dataset, RCMF did not obtain the best subset, but it achieved comparable classification performance to the other five filters.

From Table 5, it can be observed that RCMF achieved the best performance in the majority of datasets. RCMF overcomes the limitation of traditional filters by considering inter-gene correlations and taking into account redundancy and complementarity relationships between genes. This not only enhances the diversity of the filtered gene subset but also leads to significantly improved performance.

### 5.3. The Performance of the GBGPSEO Algorithm

Table 6 show the algorithm’s performance compared with nine other algorithms. To ensure fairness in the experiment, all methods involved in the comparison employed the RCMF strategy to generate experimental results. MeanA represents the average classification accuracy obtained from 20 independent experiments. MeanS represents the average size of the gene set obtained from 20 independent experiments. MeanF represents the average fitness size obtained from 20 independent experiments. Std indicates the standard deviation. All algorithms employed the same fitness function, and each dataset underwent 20 independent experiments. From the table, it can be observed that our method achieved the highest fitness value on all datasets. Additionally, the gene subsets selected by our approach were the smallest among all datasets, and the average fitness value was the best among the 15 datasets. These results demonstrate that GBGPSEO possesses stronger search capabilities compared to other algorithms, as it achieves higher classification accuracy while significantly reducing the size of the selected gene subset.

From Table 6, it can be seen that our proposed RCMF-GBGPSEO achieves the best classification accuracy in 13 out of 15 datasets, consistently achieving the lowest average gene set sizes. On the ALLAML dataset, FGNDO attains 100% accuracy, while RCMF-GBGPSEODE achieves 99.8% accuracy with an average gene set size of 5.05. The other two algorithms have sizes of 61.08 and 60.25. The gene set size of RCMF-GBGPSEODE is only 8% of the sizes of the other two algorithms, while the accuracy drops by only 0.2%. Similar results are observed for the SRBCT-4 dataset.

In the CNS dataset, the original EO obtains the highest classification accuracy of 93.8%, with an average gene set size of 73.25 after filtering. GBGPSEO after filtering, achieves the second-highest accuracy of 92%, with an average gene set size of 24.95. Despite a 1.8% decrease in accuracy, GBGPSEO reduces the gene set by 65.9%. The original EO, after filtering, achieves accuracy above 90% on all datasets and surpasses 98% accuracy on eight datasets, indicating its strong search capability.

Our proposed GBGPSEO further enhances the search capability, reducing gene set sizes while improving classification accuracy. The most significant improvement on the Colon dataset is observed on the three UCL datasets; the most significant improvement is observed on the Colon dataset, with a 2.1% accuracy increase and an 81.2% reduction in gene set size. On the other two datasets, GBGPSEO achieves higher accuracy with gene set sizes of less than 50%. On the CAR dataset, GBGPSEO achieves 98.8% accuracy with an average gene set size of 8.1, reducing the gene count by around 60% compared to the original EO. Across other microarray datasets, GBGPSEO consistently achieves higher classification accuracy with gene set sizes around 50% of the original EO’s size.

**Figure 4:**
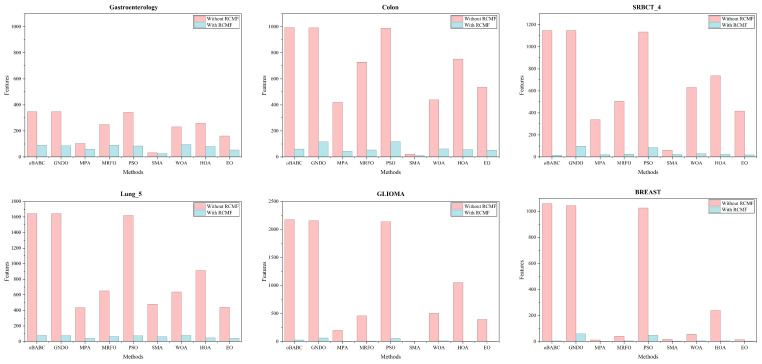
Bar chart of the comparative performance of RCMF against nine state-of-the-art algorithms.

Figure 4 reveals the performance of feature selection using the RCMF strategy across various algorithms. The red rectangles in the graph denote the number of features selected by the algorithms without the application of RCMF, while the blue rectangles represent the number of features selected after employing RCMF. It is evident from the graph that the use of the RCMF strategy significantly impacts the performance of feature selection. The bar charts illustrating the datasets demonstrate that only the SMA algorithm maintains a relatively small variation before and after the implementation of RCMF across five datasets. This reflects the superiority of the SMA algorithm in the context of feature selection challenges. Evidently, the RCMF strategy effectively filters out redundant and irrelevant features, aiding wrappers in reducing the search space while simultaneously enhancing classification accuracy.

In conclusion, the RCMF proposed in this study effectively enhances the performance of the wrapper, especially on high-dimensional and redundant datasets. GBGPSEO, while significantly reducing the gene count, also effectively improves classification accuracy, offering a more significant advantage compared to other algorithms.

### 5.4. Convergence Analysis

**Figure 5:**
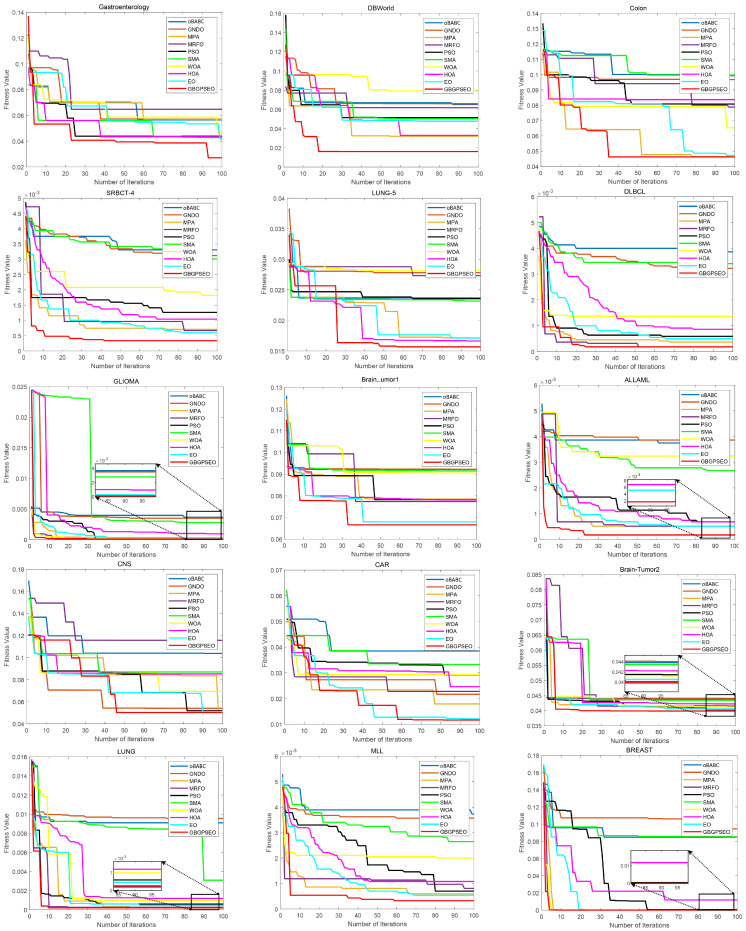
Convergence curves of different algorithms on all of the datasets.

Figure 5 displays convergence plots for ten different algorithms across fifteen datasets. All algorithms utilize the RCMF strategy with the same population size, number of iterations, and fitness function. From the graphs, it can be observed that GBGPSEO consistently exhibits the lowest convergence curves at the end of the iterations. Across fourteen datasets, GBGPS achieves the lowest convergence curves within the initial 50 iterations, indicating its strong search performance in the early stages of iteration, swiftly identifying optimal gene sets.

The original EO achieves the lowest convergence curve on the CAR dataset within the first 50 iterations. However, around the 60th iteration, GBGPSEO attains the lowest curve again, highlighting the algorithm’s superior search capabilities compared to the original EO.

In the Colon, DLBCL, GLIMA, Brain-Tumor2, LUNG, and BREAST datasets, the convergence curves of other algorithms closely resemble GBGPSEO’s. However, the corresponding table reveals that GBGPSEO still achieves the lowest fitness values. These datasets, benefiting from the RCMF strategy, produce favorable accuracies across algorithms. However, GBGPSEO’s utilization of GPS maintains high classification accuracy and significantly reduces gene set sizes, enhancing its convergence ability.

Across the remaining datasets, GBGPSEO consistently demonstrates a notable advantage over other algorithms. This indicates that GBGPSEO possesses superior convergence capabilities both in the early stages and in navigating and escaping local optima in later iterations, outperforming other algorithms.

## 6. Conclusions and Future Works

To address the limitations of conventional feature selection methods when dealing with high-dimensional microarray data, such as poor search capability and limited accuracy, this study introduces a hybrid ensemble approach called RCMF-GBGPDEO. During the filtering stage, gene correlation is considered to retain both redundant and complementary genes. The results demonstrate the method’s robust stability, significantly reducing the gene search space for subsequent gene selection methods. To enhance the search capability of the EO process, an improved Gaussian Barebone (GB) strategy combined with a gene pruning strategy (GPS) is integrated. GB enhances the EO’s ability to escape local optima in later iterations, while GPS strengthens its search capability in proximity to local optima. Experiments indicate that our method can considerably reduce the gene subset size while simultaneously improving the algorithm’s classification accuracy.

The incorporation of these two strategies leads to increased algorithmic complexity. In the future, we will explore effective methods to mitigate this complexity and enhance competitiveness. Additionally, given that the initial population is randomly initialized, we will investigate various initialization strategies to improve the initial solutions’ quality and enhance algorithm convergence speed.

In future research, we will focus on developing robust sampling strategies and feature selection techniques to mitigate the challenges posed by unbalanced datasets. Analysis of multi-omics data allows for a deeper exploration of the relationship between proposed methods and biological characteristics. In addition, we will extend our existing methods to handle multi-objective optimization problems to enhance their versatility in different application areas such as engineering design, resource allocation, and evolutionary computation.

## Figures and Tables

**Table 1:** The UCI dataset.

No.	Dataset Name	#Features	#Instances	#Classes
1	Gastroenterology	698	72	2
2	Colon	2000	62	2
3	DBWorld	4702	64	2

**Table 2:** The microarray datasets.

No.	Dataset Name	#Features	#Instances	#Classes
1	SRBCT_4	2308	83	4
2	LUNG_5	3312	203	5
3	DLBCL	4026	47	2
4	GLIOMA	4434	50	2
5	Brain_Tumor1	5920	90	5
6	ALLAML	7129	72	2
7	CNS	7130	60	2
8	CAR	9182	174	2
9	Brain_Tumor2	10,367	50	4
10	LUNG	12,533	181	2
11	MLL	12,583	72	3
12	BREAST	24,482	97	2

**Table 3:** Parameter settings of algorithms.

No.	Method	Parameter	Value
1	GBGPSEO	*a1*	2
		*a2*	1
		*GP*	0.5
		*CRmax*	1
		*CRmin*	0
		*GPP*	0.4
2	oBABC	*phiMax*	0.9
		*phiMin*	0.5
		*qStart*	0.3
		*qEnd*	0.1
3	PSO	*c1*	2
		*c2*	2
		*w*	0.5
4	WOA	*Bound*	1
5	SMA	*Z*	0.03
6	MPA	*Beta*	1.5
		*P*	0.5
		*Fad*	0.2
7	MRFO	*S*	2
8	BHOA	*w*	0.99
		*PN*	0.1
		*QN*	0.2

**Table 4:** The Jaccard index of multiple filtering thresholds.

Dataset	Top = 50	Top = 100	Top = 150	Top = 200	Top = 250	Top = 300
Gastroenterology	1	1	1	1	1	1
DBWorld	1	1	1	1	1	0.998
Colon	1	1	1	1	1	1
SRBCT_4	1	1	1	1	1	1
Lung_5	1	1	1	1	1	1
DLBCL	1	1	1	1	1	1
GLIOMA	1	1	1	1	1	1
Brain_Tumor1	1	1	1	1	1	1
ALLAML	1	1	1	1	1	1
CNS	1	1	1	1	1	1
CAR	1	1	1	1	1	1
Brain_Tumor2	1	1	1	1	1	1
LUNG	1	1	1	1	1	1
MLL	1	1	1	1	1	1
BREAST	1	1	1	1	1	1

**Table 5:** The results of RCMF compared with single filters.

Dataset	Measure	ReliefF	Fisher Score	Chi-Square	PCC	NCA	RCMF
Gastroenterology	G-mean	0.956	0.968	0.908	0.5	0.941	**0.984**
	AUC	0.927	0.938	0.833	0.5	0.885	**0.969**
DBWorld	G-mean	**0.952**	0.713	0.926	**0.952**	**0.952**	**0.952**
	AUC	**0.909**	0.591	0.864	**0.909**	**0.909**	**0.909**
Colon	G-mean	0.887	0.887	0.887	**0.935**	0.887	0.887
	AUC	0.792	0.792	0.792	**0.875**	0.792	0.792
SRBCT_4	G-mean	**1**	**1**	**1**	**1**	**1**	**1**
	AUC	**1**	**1**	**1**	**1**	**1**	**1**
Lung_5	G-mean	**0.984**	**0.984**	0.784	0.964	0.964	**0.984**
	AUC	**0.952**	**0.952**	0.85	0.904	0.904	**0.952**
DLBCL	G-mean	**1**	**1**	**1**	**1**	**1**	**1**
	AUC	**1**	**1**	**1**	**1**	**1**	**1**
GLIOMA	G-mean	0.5	**0.908**	0.5	**0.908**	0.5	**0.908**
	AUC	0.5	**0.833**	0.5	**0.833**	0.5	**0.833**
Brain_Tumor 1	G-mean	0.6	0.582	0.558	0.584	0.582	**0.774**
	AUC	0.774	0.713	0.675	0.729	0.726	**0.828**
ALLAML	G-mean	0.982	0.963	0.982	0.963	**1**	**1**
	AUC	0.964	0.929	0.964	0.929	**1**	**1**
CNS	G-mean	0.878	**0.977**	0.875	**0.977**	0.963	**0.977**
	AUC	0.786	**0.955**	0.766	**0.955**	0.929	**0.955**
CAR	G-mean	0.789	0.979	0.898	0.979	0.898	**0.99**
	AUC	0.667	0.959	0.813	0.959	0.813	**0.98**
Brain_Tumor 2	G-mean	0.96	0.874	0.96	0.96	0.96	**1**
	AUC	0.925	0.736	0.925	0.925	0.925	**1**
LUNG	G-mean	**1**	**1**	**1**	**1**	**1**	**1**
	AUC	**1**	**1**	**1**	**1**	**1**	**1**
MLL	G-mean	0.98	0.941	0.941	**0.983**	0.98	0.98
	AUC	0.959	0.884	0.884	**0.965**	0.959	0.959
BREAST	G-mean	0.91	0.869	0.871	0.5	0.929	**0.947**
	AUC	0.831	0.764	0.762	0.5	0.864	**0.898**

**Table 6:** Performance of the proposed algorithm compared with 9 other algorithms (with RCMF).

Dataset	Method	MeanA (Std)	MeanS (Std)	MeanF (Std)	Dataset	Method	MeanA (Std)	MeanS (Std)	MeanF (Std)
Gastroenterology	oBABC	0.917 (0.019)	90.5 (6.3)	0.084 (0.019)	DBWorld	oBABC	0.955 (0.013)	21.4 (4.8)	0.044 (0.007)
	GNDO	0.935 (0.016)	85.7 (7.6)	0.044 (0.011)		GNDO	0.941 (0.015)	73.0 (5.5)	0.047 (0.009)
	MPA	0.934 (0.015)	59.1 (18.2)	0.042 (0.009)		MPA	0.954 (0.013)	30.3 (10.9)	0.036 (0.009)
	MRFO	0.927 (0.020)	89.5 (26.4)	0.050 (0.010)		MRFO	0.943 (0.015)	51.1 (19.5)	0.044 (0.009)
	PSO	0.929 (0.019)	83.4 (7.5)	0.051 (0.015)		PSO	0.942 (0.021)	68.6 (6.2)	0.048 (0.011)
	SMA	0.913 (0.017)	26.1 (33.6)	0.074 (0.012)		SMA	0.924 (0.018)	17.0 (21.9)	0.074 (0.014)
	WOA	0.917 (0.015)	95.1 (41.2)	0.061 (0.012)		WOA	0.918 (0.021)	53.3 (24.2)	0.061 (0.019)
	HOA	0.811 (0.020)	79.9 (8.3)	0.082 (0.012)		HOA	0.941 (0.010)	36.7 (7.8)	0.046 (0.008)
	EO	0.933 (0.018)	52.9 (9.7)	0.043 (0.011)		EO	0.961 (0.010)	29.0 (8.4)	0.033 (0.037)
	GBGPSED	**0.935 (0.010)**	**21.7 (8.5)**	**0.031 (0.011)**		GBGPSED	**0.965 (0.015)**	**9.6 (2.9)**	**0.033 (0.012)**
Colon	oBABC	0.924 (0.015)	60.8 (8.0)	0.065 (0.006)	SRBCT_4	oBABC	0.991 (0.000)	15.4 (3.6)	0.0009 (0.000)
	GNDO	0.892 (0.010)	115.7 (6.9)	0.094 (0.008)		GNDO	**1.000 (0.000)**	97.0 (5.3)	0.0036 (0.000)
	MPA	0.914 (0.017)	43.3 (23.2)	0.066 (0.010)		MPA	0.994 (0.008)	19.7 (3.3)	0.0007 (0.000)
	MRFO	0.905 (0.019)	52.7 (38.2)	0.077 (0.014)		MRFO	0.992 (0.009)	24.9 (6.1)	0.0009 (0.000)
	PSO	0.894 (0.012)	117.8 (7.3)	0.090 (0.009)		PSO	0.996 (0.002)	84.9 (7.6)	0.0031 (0.000)
	SMA	0.908 (0.017)	10.6 (5.0)	0.077 (0.016)		SMA	0.995 (0.006)	23.6 (8.8)	0.0008 (0.000)
	WOA	0.894 (0.019)	62.0 (45.5)	0.085 (0.015)		WOA	0.991 (0.011)	30.2 (8.7)	0.0011 (0.000)
	HOA	0.938 (0.019)	56.0 (15.3)	0.067 (0.013)		HOA	0.996 (0.003)	23.0 (3.8)	0.0004 (0.000)
	EO	0.920 (0.024)	51.9 (16.1)	0.068 (0.020)		EO	0.995 (0.008)	18.6 (3.2)	0.0006 (0.000)
	GBGPSED	**0.941 (0.019)**	**9.8 (4.0)**	**0.053 (0.016)**		GBGPSED	0.998 (0.005)	**8.1 (2.1)**	**0.0003 (0.000)**
Lung_5	oBABC	0.979 (0.002)	77.4 (4.3)	0.023 (0.000)	DLBCL	oBABC	0.997 (0.004)	80.1 (3.2)	0.0036 (0.000)
	GNDO	0.978 (0.004)	76.8 (6.5)	0.022 (0.003)		GNDO	0.996 (0.004)	78.4 (4.2)	0.0035 (0.000)
	MPA	0.978 (0.005)	40.3 (14.4)	0.018 (0.002)		MPA	0.997 (0.007)	6.6 (2.5)	0.0003 (0.000)
	MRFO	0.978 (0.004)	63.2 (15.4)	0.022 (0.002)		MRFO	0.984 (0.024)	12.5 (4.5)	0.0005 (0.000)
	PSO	0.977 (0.003)	73.3 (8.9)	0.023 (0.003)		PSO	0.997 (0.004)	66.6 (5.4)	0.0030 (0.000)
	SMA	0.973 (0.005)	61.2 (28.0)	0.027 (0.002)		SMA	0.982 (0.020)	6.2 (3.2)	0.0002 (0.000)
	WOA	0.974 (0.006)	77.0 (26.8)	0.023 (0.003)		WOA	0.983 (0.015)	16.0 (9.6)	0.0007 (0.000)
	HOA	0.979 (0.004)	46.7 (9.5)	0.019 (0.002)		HOA	**0.998 (0.006)**	14.1 (3.0)	0.0006 (0.000)
	EO	0.983 (0.004)	41.1 (10.7)	0.017 (0.003)		EO	0.992 (0.013)	7.1 (2.1)	0.0003 (0.000)
	GBGPSED	**0.991 (0.004)**	**22.0 (11.1)**	**0.015 (0.004)**		GBGPSED	**0.998 (0.006)**	**4.4 (1.9)**	**0.0002 (0.000)**
GLIOMA	oBABC	0.996 (0.008)	27.1 (3.4)	0.0014 (0.000)	Brain_Tumor1	oBABC	0.929 (0.008)	24.4 (5.3)	0.080 (0.004)
	GNDO	0.995 (0.011)	62.5 (3.9)	0.0037 (0.000)		GNDO	0.911 (0.007)	82.9 (5.5)	0.088 (0.006)
	MPA	0.995 (0.011)	3.2 (1.5)	**0.0001 (0.000)**		MPA	0.921 (0.009)	20.1 (6.9)	0.080 (0.006)
	MRFO	**0.997 (0.007)**	7.7 (6.3)	0.0004 (0.000)		MRFO	0.914 (0.011)	39.2 (20.7)	0.080 (0.008)
	PSO	0.993 (0.010)	53.0 (6.2)	0.0031 (0.000)		PSO	0.911 (0.007)	75.1 (7.9)	0.088 (0.005)
	SMA	0.987 (0.015)	2.5 (0.7)	**0.0001 (0.000)**		SMA	0.903 (0.012)	21.1 (26.9)	0.090 (0.008)
	WOA	0.989 (0.012)	12.2 (15.7)	0.0007 (0.001)		WOA	0.906 (0.011)	50.3 (31.5)	0.087 (0.008)
	HOA	0.988 (0.010)	5.8 (5.3)	0.0005 (0.000)		HOA	0.903 (0.010)	39.1 (6.5)	0.088 (0.008)
	EO	0.994 (0.009)	4.7 (1.5)	0.0002 (0.000)		EO	0.923 (0.027)	18.3 (6.3)	**0.071 (0.006)**
	GBGPSED	**0.997 (0.007)**	**2.4 (0.6)**	**0.0001 (0.000)**		GBGPSED	**0.923 (0.015)**	**9.4 (3.8)**	**0.071 (0.014)**
ALLAML	oBABC	0.995 (0.000)	11.8 (1.1)	0.00086 (0.000)	CNS	oBABC	0.920 (0.021)	78.3 (8.9)	0.050 (0.010)
	GNDO	**1.000 (0.000)**	60.3 (4.7)	0.00342 (0.000)		GNDO	0.896 (0.019)	161.3 (11.1)	0.078 (0.010)
	MPA	0.996 (0.006)	8.2 (2.0)	0.00046 (0.000)		MPA	0.908 (0.017)	75.6 (38.2)	0.059 (0.014)
	MRFO	0.995 (0.006)	15.1 (7.6)	0.00086 (0.000)		MRFO	0.899 (0.019)	142.8 (37.3)	0.070 (0.011)
	PSO	0.999 (0.004)	48.6 (5.8)	0.00276 (0.000)		PSO	0.893 (0.020)	159.8 (8.9)	0.073 (0.010)
	SMA	0.991 (0.010)	8.0 (3.0)	0.00045 (0.000)		SMA	0.870 (0.028)	32.3 (48.7)	0.107 (0.013)
	WOA	0.990 (0.010)	22.4 (9.4)	0.00127 (0.001)		WOA	0.875 (0.032)	150.5 (81.0)	0.091 (0.023)
	HOA	0.992 (0.008)	9.7 (2.3)	0.00172 (0.000)		HOA	0.816 (0.022)	50.2 (13.1)	0.052 (0.019)
	EO	0.993 (0.011)	7.9 (1.8)	0.00045 (0.000)		EO	**0.938 (0.020)**	73.3 (18.9)	0.056 (0.018)
	GBGPSED	0.998 (0.005)	**5.1 (1.7)**	**0.00029 (0.000)**		GBGPSED	0.920 (0.024)	**25.0 (15.6)**	**0.046 (0.027)**
CAR	oBABC	0.978 (0.011)	30.3 (5.4)	0.023 (0.005)	Brain_Tumor2	oBABC	0.950 (0.013)	78.3 (4.8)	0.043 (0.000)
	GNDO	0.964 (0.009)	70.2 (6.7)	0.028 (0.006)		GNDO	0.950 (0.013)	72.9 (6.6)	0.043 (0.000)
	MPA	0.974 (0.007)	20.7 (16.6)	0.017 (0.005)		MPA	0.952 (0.015)	22.7 (8.5)	0.045 (0.009)
	MRFO	0.967 (0.011)	37.2 (21.3)	0.022 (0.005)		MRFO	0.937 (0.021)	43.3 (17.9)	0.041 (0.001)
	PSO	0.964 (0.010)	69.1 (4.6)	0.029 (0.006)		PSO	0.948 (0.010)	71.8 (5.6)	0.043 (0.000)
	SMA	0.967 (0.007)	9.7 (1.3)	0.027 (0.008)		SMA	0.933 (0.022)	26.4 (26.2)	0.051 (0.013)
	WOA	0.960 (0.012)	27.8 (32.2)	0.028 (0.007)		WOA	0.926 (0.021)	67.5 (31.3)	0.050 (0.009)
	HOA	0.970 (0.008)	9.4 (7.7)	0.027 (0.004)		HOA	0.933 (0.011)	33.6 (7.4)	0.051 (0.000)
	EO	0.977 (0.006)	20.3 (4.5)	0.017 (0.004)		EO	0.951 (0.012)	21.4 (5.8)	0.038 (0.006)
	GBGPSED	**0.988 (0.008)**	**8.1 (10.2)**	**0.016 (0.007)**		GBGPSED	**0.956 (0.013)**	**14.5 (10.6)**	**0.038 (0.011)**
LUNG	oBABC	0.984 (0.001)	14.2 (3.8)	0.0007 (0.000)	MLL	oBABC	0.999 (0.004)	28.5 (4.0)	0.0007 (0.000)
	GNDO	0.993 (0.003)	55.1 (4.8)	0.0100 (0.002)		GNDO	0.999 (0.003)	66.3 (5.2)	0.0035 (0.000)
	MPA	0.998 (0.003)	4.5 (1.8)	0.0002 (0.000)		MPA	0.996 (0.009)	11.2 (2.9)	0.0006 (0.000)
	MRFO	0.997 (0.004)	8.8 (4.6)	0.0011 (0.002)		MRFO	0.990 (0.011)	18.2 (7.4)	0.0009 (0.000)
	PSO	0.994 (0.003)	48.2 (5.6)	0.0093 (0.003)		PSO	0.999 (0.003)	53.5 (6.5)	0.0028 (0.000)
	SMA	0.995 (0.003)	4.2 (1.3)	0.0032 (0.003)		SMA	0.992 (0.010)	13.4 (7.1)	0.0007 (0.000)
	WOA	0.993 (0.004)	11.5 (10.1)	0.0050 (0.004)		WOA	0.988 (0.013)	27.0 (14.6)	0.0014 (0.001)
	HOA	0.998 (0.003)	11.1 (4.3)	0.0035 (0.002)		HOA	0.995 (0.006)	15.3 (3.7)	0.0008 (0.000)
	EO	0.989 (0.003)	5.4 (2.2)	0.0003 (0.000)		EO	0.999 (0.004)	11.3 (2.4)	0.0006 (0.000)
	GBGPSED	**0.999 (0.002)**	**4.0 (1.1)**	**0.0001 (0.002)**		GBGPSED	**1.000 (0.000)**	**7.5 (2.1)**	**0.0004 (0.000)**
BREAST	oBABC	**1.000 (0.000)**	4.9 (3.8)	0.00013 (0.000)					
	GNDO	**1.000 (0.000)**	58.7 (5.7)	0.00324 (0.000)					
	MPA	0.998 (0.004)	2.4 (0.6)	0.00013 (0.000)					
	MRFO	0.996 (0.007)	4.8 (2.1)	0.00026 (0.000)					
	PSO	1.000 (0.000)	48.5 (6.4)	0.00268 (0.000)					
	SMA	0.998 (0.004)	2.6 (0.6)	0.00014 (0.000)					
	WOA	0.994 (0.009)	5.6 (3.2)	0.00031 (0.000)					
	HOA	0.998 (0.003)	4.7 (1.9)	0.00017 (0.000)					
	EO	0.998 (0.004)	3.2 (1.2)	0.00017 (0.000)					
	GBGPSED	**1.000 (0.000)**	**2.1 (0.2)**	**0.00011 (0.000)**					

## Data Availability

All datasets can be found on https://archive.ics.uci.edu/ (accessed on 5 August 2025) and https://leo.ugr.es/elvira/DBCRepository/index.html (accessed on 5 August 2025).

## References

[1] Posner A., Prall O.W., Sivakumaran T., Etemadamoghadam D., Thio N., Pattison A., Balachander S., Fisher K., Webb S., Wood C. (2023). A Comparison of DNA Sequencing and Gene Expression Profiling to Assist Tissue of Origin Diagnosis in Cancer of Unknown Primary. J. Pathol..

[2] Wang J., Li X., Ma Z. (2025). Multi-Scale Three-Path Network (MSTP-Net): A New Architecture for Retinal Vessel Segmentation. Measurement.

[3] Zhao Y., Li X., Zhou C., Pen H., Zheng Z., Chen J., Ding W. (2024). A Review of Cancer Data Fusion Methods Based on Deep Learning. Inf. Fusion.

[4] Nssibi M., Manita G., Chhabra A., Mirjalili S., Korbaa O. (2024). Gene Selection for High Dimensional Biological Datasets Using Hybrid Island Binary Artificial Bee Colony with Chaos Game Optimization. Artif. Intell. Rev..

[5] Ouaderhman T., Chamlal H., Janane F.Z. (2024). A New Filter-Based Gene Selection Approach in the DNA Microarray Domain. Expert Syst. Appl..

[6] Remeseiro B., Bolon-Canedo V. (2019). A review of feature selection methods in medical applications. Comput. Biol. Med..

[7] Alhenawi E., Al-Sayyed R., Hudaib A., Mirjalili S. (2022). Feature selection methods on gene expression microarray data for cancer classification: A systematic review. Comput. Biol. Med..

[8] Salesi S., Cosma G., Mavrovouniotis M. (2021). TAGA: Tabu Asexual Genetic Algorithm embedded in a filter/filter feature selection approach for high-dimensional data. Inf. Sci..

[9] Solorio-Fernandez S., Martínez-Trinidad J.F., Carrasco-Ochoa J.A. (2020). A supervised filter feature selection method for mixed data based on spectral feature selection and information-theory redundancy analysis. Pattern Recognit. Lett..

[10] Ansari G., Ahmad T., Doja M.N. (2019). Hybrid filter–wrapper feature selection method for sentiment classification. Arab. J. Sci. Eng..

[11] Zhang R., Zhang Z. (2020). Feature selection with symmetrical complementary coefficient for quantifying feature interactions. Appl. Intell..

[12] Bommert A., Welchowski T., Schmid M., Rahnenführer J. (2022). Benchmark of filter methods for feature selection in high-dimensional gene expression survival data. Briefings Bioinform..

[13] Deng T., Chen S., Zhang Y., Xu Y., Feng D., Wu H., Sun X. (2023). A cofunctional grouping-based approach for non-redundant feature gene selection in unannotated single-cell RNA-seq analysis. Briefings Bioinform..

[14] Xu Y., Li X., Li Q. (2023). A discrete teaching–learning based optimization algorithm with local search for rescue task allocation and scheduling. Appl. Soft Comput..

[15] Nadimi-Shahraki M.H., Zamani H., Mirjalili S. (2022). Enhanced whale optimization algorithm for medical feature selection: A COVID-19 case study. Comput. Biol. Med..

[16] Mirjalili S., Mirjalili S. (2019). Genetic algorithm. Evolutionary Algorithms and Neural Networks: Theory and Applications.

[17] Vivekanandan T., Iyengar N.C.S.N. (2017). Optimal feature selection using a modified differential evolution algorithm and its effectiveness for prediction of heart disease. Comput. Biol. Med..

[18] Almasoudy F.H., Al-Yaseen W.L., Idrees A.K. (2020). Differential evolution wrapper feature selection for intrusion detection system. Procedia Comput. Sci..

[19] Chen H., Li S., Li X., Zhao Y., Dong J. (2023). A hybrid adaptive Differential Evolution based on Gaussian tail mutation. Eng. Appl. Artif. Intell..

[20] Sowan B., Eshtay M., Dahal K., Qattous H., Zhang L. (2023). Hybrid PSO Feature Selection-Based Association Classification Approach for Breast Cancer Detection. Neural Comput. Appl..

[21] Ghosh M., Guha R., Sarkar R., Abraham A. (2020). A wrapper-filter feature selection technique based on ant colony optimization. Neural Comput. Appl..

[22] Ma W., Zhou X., Zhu H., Li L., Jiao L. (2021). A two-stage hybrid ant colony optimization for high-dimensional feature selection. Pattern Recognit..

[23] Fu Q., Li Q., Li X. (2023). An improved multi-objective marine predator algorithm for gene selection in classification of cancer microarray data. Comput. Biol. Med..

[24] Meenachi L., Ramakrishnan S. (2020). Differential evolution and ACO based global optimal feature selection with fuzzy rough set for cancer data classification. Soft Comput..

[25] Hu J., Gui W., Heidari A.A., Cai Z., Liang G., Chen H., Pan Z. (2022). Dispersed foraging slime mould algorithm: Continuous and binary variants for global optimization and wrapper-based feature selection. Knowl.-Based Syst..

[26] Qiu F., Guo R., Chen H., Liang G. (2022). Boosting Slime Mould Algorithm for High-Dimensional Gene Data Mining: Diversity Analysis and Feature Selection. Comput. Math. Methods Med..

[27] Hu J., Chen H., Heidari A.A., Wang M., Zhang X., Chen Y., Pan Z. (2021). Orthogonal learning covariance matrix for defects of grey wolf optimizer: Insights, balance, diversity, and feature selection. Knowl.-Based Syst..

[28] Samieiyan B., MohammadiNasab P., Mollaei M.A., Hajizadeh F., Kangavari M. (2022). Novel optimized crow search algorithm for feature selection. Expert Syst. Appl..

[29] Xu Y., Li X., Li Q., Zhang W. (2023). An improved estimation of distribution algorithm for rescue task emergency scheduling considering stochastic deterioration of the injured. Complex & Intelligent Systems.

[30] Tsai C.F., Sung Y.T. (2020). Ensemble feature selection in high dimension, low sample size datasets: Parallel and serial combination approaches. Knowl.-Based Syst..

[31] Abeel T., Helleputte T., Van de Peer Y., Dupont P., Saeys Y. (2010). Robust biomarker identification for cancer diagnosis with ensemble feature selection methods. Bioinformatics.

[32] Singh N., Singh P. (2021). A hybrid ensemble-filter wrapper feature selection approach for medical data classification. Chemom. Intell. Lab. Syst..

[33] Lai C.M., Yeh W.C., Chang C.Y. (2016). Gene selection using information gain and improved simplified swarm optimization. Neurocomputing.

[34] Mandal M., Singh P.K., Ijaz M.F., Shafi J., Sarkar R. (2021). A tri-stage wrapper-filter feature selection framework for disease classification. Sensors.

[35] Sun Y., Lu C., Li X. (2018). The cross-entropy based multi-filter ensemble method for gene selection. Genes.

[36] Drotár P., Gazda M., Vokorokos L. (2019). Ensemble feature selection using election methods and ranker clustering. Inf. Sci..

[37] Yao G., Hu X., Wang G. (2022). A novel ensemble feature selection method by integrating multiple ranking information combined with an SVM ensemble model for enterprise credit risk prediction in the supply chain. Expert Syst. Appl..

[38] Thejas G., Garg R., Iyengar S.S., Sunitha N., Badrinath P., Chennupati S. (2021). Metric and accuracy ranked feature inclusion: Hybrids of filter and wrapper feature selection approaches. IEEE Access.

[39] Wang A., Liu H., Yang J., Chen G. (2022). Ensemble feature selection for stable biomarker identification and cancer classification from microarray expression data. Comput. Biol. Med..

[40] Chiew K.L., Tan C.L., Wong K., Yong K.S., Tiong W.K. (2019). A new hybrid ensemble feature selection framework for machine learning-based phishing detection system. Inf. Sci..

[41] Ngo G., Beard R., Chandra R. (2022). Evolutionary bagging for ensemble learning. Neurocomputing.

[42] Faramarzi A., Heidarinejad M., Stephens B., Mirjalili S. (2020). Equilibrium optimizer: A novel optimization algorithm. Knowl.-Based Syst..

[43] Rizk-Allah R.M., Hassanien A.E. (2023). A hybrid equilibrium algorithm and pattern search technique for wind farm layout optimization problem. ISA Trans..

[44] Sun Y., Pan J.S., Hu P., Chu S.C. (2023). Enhanced equilibrium optimizer algorithm applied in job shop scheduling problem. J. Intell. Manuf..

[45] Seleem S.I., Hasanien H.M., El-Fergany A.A. (2021). Equilibrium optimizer for parameter extraction of a fuel cell dynamic model. Renew. Energy.

[46] Soni J., Bhattacharjee K. (2024). Multi-Objective Dynamic Economic Emission Dispatch Integration with Renewable Energy Sources and Plug-in Electrical Vehicle Using Equilibrium Optimizer. Environ. Dev. Sustain..

[47] Abdel-Basset M., Chang V., Mohamed R. (2021). A novel equilibrium optimization algorithm for multi-thresholding image segmentation problems. Neural Comput. Appl..

[48] Houssein E.H., Dirar M., Abualigah L., Mohamed W.M. (2022). An efficient equilibrium optimizer with support vector regression for stock market prediction. Neural Computing and Applications.

[49] Zhong C., Li G., Meng Z., Li H., He W. (2023). A self-adaptive quantum equilibrium optimizer with artificial bee colony for feature selection. Comput. Biol. Med..

[50] Thapliyal S., Kumar N. (2024). ASCAEO: Accelerated Sine Cosine Algorithm Hybridized with Equilibrium Optimizer with Application in Image Segmentation Using Multilevel Thresholding. Evol. Syst..

[51] Agrawal U., Rohatgi V., Katarya R. (2022). Normalized mutual information-based equilibrium optimizer with chaotic maps for wrapper-filter feature selection. Expert Syst. Appl..

[52] Guha R., Ghosh K.K., Bera S.K., Sarkar R., Mirjalili S. (2023). Discrete equilibrium optimizer combined with simulated annealing for feature selection. J. Comput. Sci..

[53] Gu Q., Li Z., Han J. (2012). Generalized fisher score for feature selection. arXiv.

[54] Urbanowicz R.J., Meeker M., La Cava W., Olson R.S., Moore J.H. (2018). Relief-based feature selection: Introduction and review. J. Biomed. Inform..

[55] Su C.T., Hsu J.H. (2005). An extended chi2 algorithm for discretization of real value attributes. IEEE Trans. Knowl. Data Eng..

[56] Benesty J., Chen J., Huang Y. (2008). On the importance of the Pearson correlation coefficient in noise reduction. IEEE Trans. Audio Speech Lang. Process..

[57] Tuncer T., Ertam F. (2020). Neighborhood component analysis and reliefF based survival recognition methods for Hepatocellular carcinoma. Phys. A Stat. Mech. Its Appl..

[58] Al-Betar M.A., Abu Doush I., Makhadmeh S.N., Al-Naymat G., Alomari O.A., Awadallah M.A. (2024). Equilibrium Optimizer: A Comprehensive Survey. Multimed. Tools Appl..

[59] Houssein E.H., Hassan M.H., Mahdy M.A., Kamel S. (2023). Development and Application of Equilibrium Optimizer for Optimal Power Flow Calculation of Power System. Appl. Intell..

[60] Witten I.H., Frank E., Trigg L., Hall M., Holmes G., Cunningham S.J. (1999). Weka: Pracitcal Machine Learning Tools and Techniques with Java Implementations.

[61] Koller D., Sahami M. Toward optimal feature selection. Proceedings of the ICML.

[62] Haque M.N., Sharmin S., Ali A.A., Sajib A.A., Shoyaib M. (2021). Use of Relevancy and Complementary Information for Discriminatory Gene Selection from High-Dimensional Gene Expression Data. PLoS ONE.

[63] Wang H., Rahnamayan S., Sun H., Omran M.G. (2013). Gaussian bare-bones differential evolution. IEEE Trans. Cybern..

[64] Lai C.M. (2019). Integrating simplified swarm optimization with AHP for solving capacitated military logistic depot location problem. Appl. Soft Comput..

[65] Li T., Zhang C., Ogihara M. (2004). A comparative study of feature selection and multiclass classification methods for tissue classification based on gene expression. Bioinformatics.

[66] Wang Z., Ning X., Blaschko M. (2023). Jaccard Metric Losses: Optimizing the Jaccard Index with Soft Labels. Adv. Neural Inf. Process. Syst..

[67] Eelbode T., Bertels J., Berman M., Vandermeulen D., Maes F., Bisschops R., Blaschko M.B. (2020). Optimization for Medical Image Segmentation: Theory and Practice When Evaluating With Dice Score or Jaccard Index. IEEE Trans. Med. Imaging.

[68] Ri J.H., Tian G., Liu Y., Xu W.h., Lou J.g. (2020). Extreme Learning Machine with Hybrid Cost Function of G-mean and Probability for Imbalance Learning. Int. J. Mach. Learn. Cybern..

[69] Góra G., Skowron A., Campagner A., Urs Lenz O., Xia S., Ślęzak D., Wąs J., Yao J. (2023). On kNN Class Weights for Optimising G-Mean and F1-Score. Proceedings of the Rough International Joint Conference on Rough Sets.

[70] Zhu F., Shuai Z., Lu Y., Su H., Yu R., Li X., Zhao Q., Shuai J. (2024). oBABC: A One-Dimensional Binary Artificial Bee Colony Algorithm for Binary Optimization. Swarm Evol. Comput..

[71] Qu L., He W., Li J., Zhang H., Yang C., Xie B. (2023). Explicit and Size-Adaptive PSO-based Feature Selection for Classification. Swarm Evol. Comput..

[72] Liu W., Guo Z., Jiang F., Liu G., Wang D., Ni Z. (2022). Improved WOA and Its Application in Feature Selection. PLoS ONE.

[73] Ewees A.A., Ismail F.H., Sahlol A.T. (2023). Gradient-Based Optimizer Improved by Slime Mould Algorithm for Global Optimization and Feature Selection for Diverse Computation Problems. Expert Syst. Appl..

[74] Asghari Varzaneh Z., Hosseini S., Javidi M.M. (2023). A Novel Binary Horse Herd Optimization Algorithm for Feature Selection Problem. Multimed. Tools Appl..

[75] Alrasheedi A.F., Alnowibet K.A., Saxena A., Sallam K.M., Mohamed A.W. (2022). Chaos Embed Marine Predator (CMPA) Algorithm for Feature Selection. Mathematics.

[76] Amin J., Anjum M.A., Ahmad A., Sharif M.I., Kadry S., Kim J. (2024). Microscopic Parasite Malaria Classification Using Best Feature Selection Based on Generalized Normal Distribution Optimization. PeerJ Comput. Sci..

[77] Got A., Zouache D., Moussaoui A., Abualigah L., Alsayat A. (2024). Improved Manta Ray Foraging Optimizer-based SVM for Feature Selection Problems: A Medical Case Study. J. Bionic Eng..

[78] Abdulrauf Sharifai G., Zainol Z. (2020). Feature selection for high-dimensional and imbalanced biomedical data based on robust correlation based redundancy and binary grasshopper optimization algorithm. Genes.

